# *In vitro* exercise model using contractile human and mouse hybrid myotubes

**DOI:** 10.1038/s41598-019-48316-9

**Published:** 2019-08-15

**Authors:** Weijian Chen, Mazvita R. Nyasha, Masashi Koide, Masahiro Tsuchiya, Naoki Suzuki, Yoshihiro Hagiwara, Masashi Aoki, Makoto Kanzaki

**Affiliations:** 1 Graduate School of Biomedical Engineering, Tohoku University, 980-8579, 6-6-04 Aoba, Aramaki, Aoba-ku, Sendai, Japan; 2 Department of Orthopaedic Surgery, Graduate School of Medicine, Tohoku University, 980-8575, Sendai, Japan; 3 Department of Nursing, Tohoku Fukushi University, 981-8522, Sendai, Japan; 4 Department of Neuroscience, Tohoku University Graduate School of Medicine, 980-8575, Sendai, Japan

**Keywords:** Metabolic syndrome, Cell culture

## Abstract

Contraction of cultured myotubes with application of electric pulse stimulation (EPS) has been utilized for investigating cellular responses associated with actual contractile activity. However, cultured myotubes derived from human subjects often exhibit relatively poor EPS-evoked contractile activity, resulting in minimal contraction-inducible responses (*i*.*e*. myokine secretion). We herein describe an “*in vitro* exercise model”, using hybrid myotubes comprised of human myoblasts and murine C2C12 myoblasts, exhibiting vigorous contractile activity in response to EPS. Species-specific analyses including RT-PCR and the BioPlex assay allowed us to separately evaluate contraction-inducible gene expressions and myokine secretions from human and mouse constituents of hybrid myotubes. The hybrid myotubes, half of which had arisen from primary human satellite cells obtained from biopsy samples, exhibited remarkable increases in the secretions of human cytokines (myokines) including interleukins (IL-6, IL-8, IL-10, and IL16), CXC chemokines (CXCL1, CXCL2, CXCL5, CXCL6, CXCL10), CC chemokines (CCL1, CCL2, CCL7, CCL8, CCL11, CCL13, CCL16, CCL17, CCL19, CCL20, CCL21, CCL22, CCL25, CCL27), and IFN-γ in response to EPS-evoked contractile activity. Together, these results indicate that inadequacies arising from human muscle cells are effectively overcome by fusing them with murine C2C12 cells, thereby supporting the development of contractility and the resulting cellular responses of human-origin muscle cells. Our approach, using hybrid myotubes, further expands the usefulness of the “*in vitro* exercise model”.

## Introduction

“*In vitro* exercise models”, using myotubes contracting in response to the application of electric pulse stimulation (EPS)^1^, have been widely utilized for investigating the impacts of actual contractile activity on muscle cell properties including exercise-inducible myokine secretion^2^, hypertrophy^3^, sarcomere lesions^4^ and beneficial anti-lipotoxicity effects^5^. Although most prior studies used murine muscle cell lines such as mouse C2C12 cells^6–8^, several investigations have also been conducted using human muscle cells and provided compelling evidence that “*in vitro* exercise models” are potentially applicable not only to murine muscle cells but also muscle cells obtained from human subjects^3,9–15^.

Primary myoblasts (activated satellite cells) obtained from human subjects behave in essentially the same way as murine myoblasts in cell cultures and they usually undergo several rounds of cell division until reaching confluence in growth media, followed by terminal differentiation that eventually resulted in the formation of multinuclear myotubes via successive cellular fusions under low-serum conditions in differentiation media^16^. However, primary human myotubes often exhibited poorer contractile activity than mouse C2C12 myotubes in response to EPS due to several intrinsic traits of human-derived muscle cells. These traits include the slower growth rate of myoblasts accompanied by a limitation in reaching full-confluence as well as the markedly flattened myotubular morphology reflecting their firm adhesion to the substratum. In addition, it is difficult to obtain large numbers of satellite cells from skeletal muscle biopsies of patients, and the primary satellite cells, especially those obtained from elderly patients, generally exhibit a restricted proliferative capability even in culture, which often makes it more problematic to obtain sufficient numbers of myoblasts (activated satellite cells) for producing myotubes suitably applicable to the “*in vitro* exercise model”.

In this study, taking advantage of the features of vital cellular fusion in myogenic differentiation, we documented that hybrid myotubes comprised of human-origin myoblasts and murine-origin C2C12 myoblast cells effectively acquired vigorous contractile activity in response to EPS. This acquisition was obviously attributable to the supportive functions of C2C12 in ameliorating the aforementioned adverse characteristics of primary human muscle cells. Importantly, contraction-dependent mRNA upregulations of myokines from human-origin nuclei of hybrid myotubes were detected utilizing species-specific PCR primer sets. Moreover, the Bioplex assay for human cytokines/interleukins demonstrated that EPS-evoked contraction markedly stimulates secretion of a wide array of human myokines from the hybrid myotubes. Thus, we propose that an “*in vitro* exercise model” using human and murine hybrid myotubes is a potentially valuable alternative method for analyzing contractility and its associated biological responses in human-origin muscle cells, which would allow us to maximize the use of the muscle cells obtained from biopsy samples, a very limited resource.

## Materials and Methods

### Materials

Dulbecco’s modified Eagle’s medium (DMEM), penicillin/streptomycin, and trypsin-EDTA were purchased from Sigma Chemical (St. Louis, MO). Cell culture equipment and rectangular 8-well plates were obtained from BD Biosciences (San Jose, CA) and ThermoFisher Scientific (Rochester, NY, USA), respectively. Calf serum and fetal bovine serum (FBS) were obtained from BioWest (Nuaille, France). The Transcriptor First Strand cDNA Synthesis Kit and LighCycler480 qRT-PCR reagents were purchased from ThermoFisher Scientific (Rochester, NY, USA). Matrigel was obtained from Corning (#354230, NY, USA). Unless otherwise noted, all chemicals were of the purest grade available from Sigma Chemical or Wako Pure Chemical Industries (Osaka, Japan).

### Cell culture

Mouse skeletal muscle cells, C_2_C_12_ myoblasts (passages no. 4–10)^17^, were maintained in Dulbecco’s Modified Eagle’s Medium (DMEM; Wako Pure Chemicals Industries, Osaka, Japan) containing 4.5 g/l glucose supplemented with 20% FBS, 30 μg/ml penicillin, and 100 μg/ml streptomycin (growth medium) at 37 °C under a 5% CO_2_ atmosphere. Human skeletal muscle myoblasts (HSMM) (Cat.# CC-2580) were purchased from Lonza (Walkersville, MD) and were cultured following the vendor’s instructions. C2C12 alone, or HSMM alone, were seeded at a density of 1.25 × 10^5^ cells/well in 3 ml of growth medium in 8-well plates (Nalgen Nunc International, Rochester, NY). For mixed cultures, C2C12 cells (0.625 × 10^5^ cells) and HSMM cells (0.625 × 10^5^ cells) were blended in advance at a final density of 1.25 × 10^5^ cells/well in total and were seeded onto 8-well plates. In some experiments, human satellite cells obtained by FACS (see below) were used instead of the HSMM cells. Three days after plating, the cells had reached 80–90% confluence (*day 0*). Differentiation was then induced by switching the growth medium to DMEM supplemented with 2% horse serum, 30 μg/ml penicillin, and 100 μg/ml streptomycin (differentiation medium). The differentiation medium was changed every 24 h during the 7–8 days of differentiation.

For immunofluorescent analysis, C2C12 alone or HSMM alone were seeded at a density of 1.5 × 10^4^ cells/well in 1 ml of growth medium on Matrigel-coated Cell-Disk LF (Sumitomo Bakelite Co. LTD, Japan) in a 24-well plate. For mixed culture, C2C12 cells (0.75 × 10^4^ cells) and HSMM cells (0.75 × 10^4^ cells) were blended in advance at a final density of 1.5 × 10^4^ cells/well in total in 1 mL of growth medium and were seeded onto Matrigel-coated Cell-Disk LF in a 24-well plate. In some mixed culture experiments, C2C12 cells constitutively expressing GLUT4-ECFP (GLUT4-ECFP-C2C12)^18^, instead of C2C12 cells, were used for generating hybrid myotubes. Three days after plating, these cells had reached 80–90% confluence, and myogenic differentiation was induced by replacing the growth medium with differentiation medium. The differentiation medium was changed every 24 h during the 7–8 days of differentiation.

### Human satellite cell isolation and proliferation

Human satellite cells were isolated from intact subscapularis muscles of patients who agreed to undergo biopsy during surgical repair of arthroscopic rotator cuff tears (RCT), as previously reported^16^. This study was approved by the Tohoku University Hospital Institutional Review Board (approval number: 2014-1-703), and written informed consent was obtained from all participants. All experiments were performed in accordance with relevant guidelines and regulations. In this study, the intact subscapularis, but not the disused supraspinatus (the ruptured supraspinatus tendon), muscle of a 63-year-old male patient was subjected to further *in vitro* experiments. Briefly, the tissue was minced and digested with 0.2% collagenase (Wako Pure Chemicals Industries) and 0.1% DNase I (Sigma-Aldrich, St. Louis, MO, USA), filtered through a 70-μm cell strainer (BD Biosciences, Franklin Lakes, NJ, USA) and centrifuged at 700 x *g* for 20 min. Pellets were resuspended in phosphate buffered saline (PBS) containing 1% bovine serum albumin (BSA; Sigma-Aldrich) and then incubated with an Fc receptor blocking solution (Human TruStain FcX, 1:20 in staining buffer; Biolegend, San Diego, CA, USA). The samples were then labeled with the following monoclonal antibodies (all from Biolegend and all at 1:20 dilution): fluorescein isothiocyanate (FITC)-conjugated anti-CD45 (clone HI30), FITC-conjugated anti-CD11b (clone ICRF444), FITC-conjugated anti-CD31 (clone WM59), phycoerythrin (PE)/Cy7-conjugated anti-CD34 (clone 581), allophycocyanin (APC)-conjugated anti-CD56 (clone MEM-188), and PE-conjugated anti-PDGFR*α* (clone 16A1). The negative set included blood markers CD11b and CD45, and endothelial markers CD31 and CD34. Although CD34 is known to be expressed by the majority of mouse satellite cells^19^, human muscle-derived CD34+ cells are myogenic and adipogenic, whereas CD34− cells are myogenic but not adipogenic^20^. Therefore, CD34 was used as a negative selection marker. Human satellite cells were defined as single live mononuclear CD11b^−^CD31^−^CD34^−^CD45^−^CD56^+^ cells. Fluorescence-activated cell sorting (FACS) was performed on a FACS ARIA II flow cytometer (BD Biosciences). Cells were seeded onto 24-well chamber slides coated with Matrigel (Dow Corning, Corning, NY, USA) in a growth medium containing DMEM/Ham’s F10 mixture supplemented with 20% FBS, 1% penicillin-streptomycin, 1% chicken embryonic extract (United States Biological, Salem, MA, USA), and 2.5-ng/ml basic fibroblast growth factor (Thermo Fischer Scientific, Waltham, MA, USA) and cultured at 37 °C in a 5% CO_2_ atmosphere. When cells reached 60%-80% confluence, adherent cells were dissociated and split onto a new Matrigel-coated 15-cm dish to expand the activated satellite cells. Activated satellite cells (myoblasts) were suspended in Cell Banker (TAKARA, CB011, Japan) and stored in liquid nitrogen.

### Immunofluorescence analysis

After the experimental treatments, cells were washed with PBS and fixed for 20 min with 2% paraformaldehyde in PBS containing 0.1% Triton X-100, then washed and blocked in PBS containing 5% calf serum with 1% BSA for 1 h at room temperature. For immunofluorescence analysis, anti-human nuclear antigen antibody (MAB4470; R&D Systems, Minneapolis, MN, USA) and anti-caveolin 3 antibody (PA1-066, Affinity BioReagents, CO, USA) as the first antibodies, and Alexa Fluor 488-conjugated anti-mouse IgG and Alexa Flour 594-conjugated anti-rabbit IgG as the secondary antibodies (Thermo Fischer Scientific) were used at 1:100 and 1:1000 dilutions, respectively, in a solution of 1% BSA in PBS. For analyzing the hybrid myotubes derived from a mixed culture of HSMM and GLUT4-ECFP-C2C12 cells, anti-human nuclear antibody as the first antibody and Alexa Fluor 594-conjugated anti-mouse IgG as the secondary antibody were used. The samples were mounted on glass slides with Vectashield (Vector Laboratories, Burlingame, CA, USA) and observed with a confocal fluorescence microscope (Fluoview FV-1000; Olympus, Tokyo, Japan) with an oil-immersion objective lens (PLANPON60xOSC2, NA 1.4, Olympus) and ASW v.1.3 software (Olympus). The fluorescence of DAPI, Alexa 488, and Alexa 555 was excited at 405, 488, and 543 nm laser wavelengths and detected through BA430-470, BA505-525, and BA560–600 nm bandpass filters, respectively. The fluorescence of ECFP was excited at a 405 nm laser wavelength and detected through a BA465-495 nm bandpass filter. The pinhole diameters (confocal aperture) are set automatically according to the selected dyeing method, and imaging was performed at 8.0–10 us/pixel (Scan speed) for 1024 × 768 (4:3) or 1024 × 1024 (1:1) pixels applying a sequential scan mode with Kalman filtering. The number of HNA-positive nuclei and the total number of nuclei were counted in each field, and human nuclei were calculated as the ratio of HNA-positive nuclei vs. the total number of nuclei. Images were imported into Adobe Photoshop 6.0 (Adobe Systems, San Jose, CA, USA) for processing. Three independent experiments were performed under each condition.

### Electrical pulse stimulation

Differentiated myotubes in 8-well dishes (Nalgen Nunc International) or on Cell-Disk LF were placed in a chamber for electrical stimulation (C-Dish; IonOptix, Milton, MA). Electrical stimulation (1 Hz, 4-ms, 20 V/25 mm) was applied to the cells in the C-Dish using a C-Pace pulse generator (C-Pace 100; IonOptix). DMEM containing 2% calf serum supplemented with 200% amino acids (Sigma) was used during the EPS treatments, as previously reported^1^. In the present study, we applied 16 h of EPS treatment and then harvested the conditioned media and the cells for analyses, since a longer time (~12 h) was required for the induction of IL-6 after the initiation of ESP as compared to other contraction-inducible CXCL1, which showed induction within 3 h in our *in vitro* exercise model using C2C12 cells^2,21^.

### Calculation of movement index

For evaluating the EPS-evoked contractile activity of myotubes, the index of movement was calculated by the differential image subtraction method with a slight modification, as we reported previously^22^. Briefly, high-quality images of cells were taken sequentially with a CCD camera (Hamamatsu C3077-70, Japan) and a dissection microscope (Olympus CKX41) equipped with a 20X objective lens at 14.4 fps during EPS. We usually obtained the movies at the end (the last ~15 min) of 16 h EPS treatment for the contractile activity evaluations. The image showing the first maximum contraction state of the myotubes was used as the starting point, and the images obtained over the next 3–5 seconds were used for analyzing contractile activity. The images for each second correspond to a contraction period, and the images in between them (e.g. those obtained at approximately 0.5 seconds after the first image) were used as the relaxation period for the 1 Hz EPS experiment. The images of the myotubes in the maximum contraction state and the relaxation state in each second were used for making the differential images. The differential images were obtained by subtracting the relaxation image from the contraction image. Given that the difference in pixel intensity between the first (contraction) and successive (relaxation) images is due to changes in the movement of scattering objects, the overlaid images of these subtracted images indicate moving parts. The calculated average intensity of the differential image indicates the amount of myotube movement (movement index). Three different fields under each culture condition were used for evaluation of the movement index.

### Real-time PCR

Total RNA was extracted from cell cultures using TRIzol reagent (Molecular Research Center Inc., Cincinnati, OH, USA). cDNA was synthesized using a Transcriptor First Strand cDNA Synthesis Kit with oligo-dT primers (Roche Diagnostics, Indianapolis, IN, USA). Then, fluorescence real-time PCR analysis was performed using a Light Cycler 480 II instrument and SYBR Green detection kit according to the manufacturer’s protocol (Roche). The relative mRNA expression levels of the target genes were calculated using mouse glyceraldehyde 3-phosphate dehydrogenase (GAPDH) and human 60 S acidic ribosomal protein p0 (RPLP0) as a reference, respectively. PCR primers for measuring each of the secreted factors were as follows: for mouse CXCL1/KC, 5 -GCT GGC TTC TGA CAA CAC TAT-3 and 5 -CAA GCA GAA CTG AAC TAC CAT- 3; for mouse IL6, 5-CAA TGC TCT CCT AAC AGA TAA G-3 and 5-AGG CAT AAC GCA CTA GGT-3; for mouse GAPDH, 5-GGA GAA ACC TGC CAA GTA TGA-3, 5- GCA TCG AAG GTG GAA GAG T-3; for human CXCL1, 5-GCT TGC CTC AAT CCT GCA TC-3 and 5-GGT CAG TTG GAT TTG TCA CTG T-3; for human IL6, 5-ATC TGG ATT CAA TGA GGA GAC T-3 and 5-TGT TCC TCA CTA CTC TCA AAT CTG-3; for human RPLP0, 5-GGA AAC TCT GCA TTC TCG CT-3 and 5-GCA AGT GGG AAG GTG TAA TCC-3.

### Bio-plex assay

Levels of human cytokines/interleukins in culture supernatants were measured with commercially available BioPlex kits (Bio-Rad Laboratories, Cat#. 171-AK99MR2). The mouse CXCL1 and IL-6 concentrations were measured employing a house-made BioPlex assay with antibodies from the commercially available ELISA kits (R & D Systems, Minneapolis, MN).

### Statistical analysis

Statistical analyses were performed using Student’s *t*-test, and *P* values < 0.05 were considered to indicate a statistically significant difference. Data are expressed as means ± SE unless otherwise specified.

## Results

### Human myoblasts and mouse C2C12 myoblasts undergo successive fusions with each other and to form hybrid myotubes

**Figure 1:**
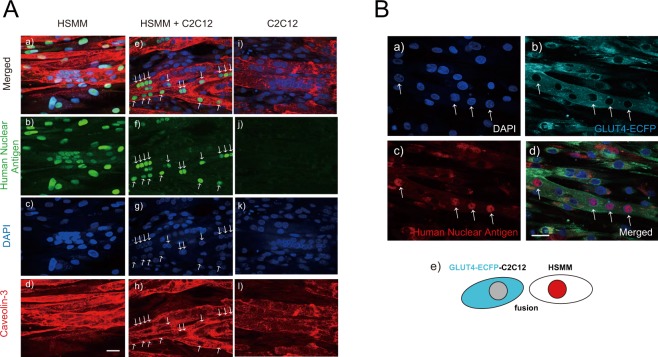
Status of fusion between human and mouse muscle cells. (**A**) After 7–8 days of differentiation, the differentiated myotubes derived from human alone (HSMM, *panels a–d*), human-origin and mouse-origin cells in a mixture (HSMM + C2C12, *panels e–h*), and murine C2C12 cells alone (*panels i–l*) were fixed and then analyzed for myotubular fusion status by using anti-human nuclear antigen (anti-HNA) and anti-Caveolin 3 antibodies, as described in the Materials and Methods. DAPI was used for nuclear staining. Scale bar = 25 μm. Three independent experiments were performed and representative images are presented. Note that both anti-HNA-positive and -negative nuclei are observed in a myotube from the mixed culture (HSMM + C2C12) (*panels e*–*g*). (**B**) Differentiated myotubes that arose from HSMM and GLUT4-ECFP-expressing C2C12 cells (*panel e*) were fixed and then analyzed for their myotubular fusion status by using GLUT4-ECFP (*panel b*) and anti- HNA antibody (*panel c*). Three independent experiments were performed and representative images are presented. Note that the myotube expressing GLUT4-ECFP (*panel b*) contains anti-HNA-positive nuclei (*panel d*, *allows*). DAPI was used for nuclear staining (*panel a*). Scale bar = 25 μm.

To establish an *in vitro* exercise model using myotubes hybridized from human myoblasts and the mouse C2C12 cell line, we first studied the myogenic differentiation status of human cells alone, mouse C2C12 cells alone, and a mixture of the two (1:1) in cultures by immunofluorescent staining using anti-human nuclear antigen (HNA) antibody for specifying human-derived HSMM cells and anti-cavelin-3 antibody for evaluating myogenic differentiation and fusogenic status (Fig. 1A). Anti-HNA antibody faithfully detected only the nuclei of human cell origin in the HSMM alone group (*panel b*), i.e. not those of murine C2C12 cells (*panel j*). In the mixed culture of both HSMM and C2C12 cells, anti-HNA antibody detected several, but not all, nuclei (*panels e* and *f*) of the multi-nucleated myotubes expressing caveolin-3, a myogenic differentiation marker protein (*panel h*), indicating that both HNA-positive nuclei derived from HSMM cells and NHA-negative nuclei derived from murine C2C12 cells existed in a single myotube. Even though the same numbers of HSMM (0.75 × 10^4^ cells) and C2C12 cells (0.75 × 10^4^ cells) had initially been seeded, the proportion of human nuclei decreased to 14.25 ± 1.52% (seven separate fields, each containing 67–140 total nuclei, were counted) in the hybrid myotubes at Day7 of differentiation, probably due to the slower growth rate of HSMM than C2C12 cells.

To further confirm the hybrid myotube formation between human and murine myoblastic cells, a C2C12 myoblast cell line constitutively expressing GLUT4-ECFP^18^, rather than wild type C2C12 myoblasts, was subjected to the mixed culture experiment (Fig. 1B). As expected, HSMM cells underwent successive fusions with murine C2C12 expressing GLUT4-ECFP to form GLUT4-ECFP-positive myotubes (*panel b*) harboring many anti-HNA-positive nuclei (*panel c*). Together, these data obtained from two distinct sets of experiments demonstrated that human myoblasts and murine C2C12 cells had fused with each other to form hybrid multi-nucleated myotubes.

### Human and murine hybrid myotubes exhibited vigorous contractile activity in response to EPS

**Figure 2:**
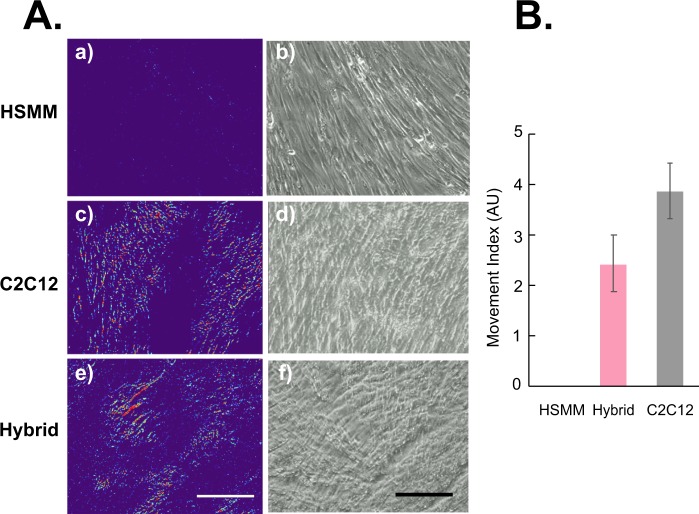
Evaluation of contractile activity of cultured myotubes. After 7–8 days of differentiation, EPS (20 V/25 mm, 1-Hz frequency, 4-ms duration) was applied to differentiated myotubes originating from human samples alone (HSMM, *panels a and b*), murine C2C12 cells alone (*panels c and d*), and a mixture of the two (hybrid, *panels e and f*) for 16 h. Images of myotubes were then taken sequentially during EPS at the last 15 min. of the total EPS session (Supplementary Movies S1–S3). (**A**) The pseudo-colored differential images (*panels a*, *c and e*), reflecting contractile area and ability, were produced as described in the Materials and Methods. The bright-field images of the same area (*panels b*, *d*, *and f*) are also presented. Scale bar = 250 μm. Three independent experiments were performed and representative images are presented. (**B**) The Movement Index was determined as described in the Materials and Methods (*n* = 3; *P < 0.05).

As shown in Fig. 1A, myogenic differentiation and myotube formation were similarly achieved under HSMM alone, HSMM + C2C12, and C2C12 alone culture conditions. However, HSMM myotubes exhibited minimal ability to acquire EPS-evoked contractile activity under this culture condition (Fig. 2, *panel a*), despite delivery of the same EPS that had effectively endowed C2C12 myotubes with vigorous contraction capability (*panel c*) as previously reported^1^. Intriguingly, however, hybrid myotubes arising from both human and murine myoblasts acquired significant contractile ability (*panel e*) at levels similar to those of the C2C12 myotubes in response to the same intensity of EPS **(**Fig. 2B) (Supplementary Movies S1–S3). These results clearly indicate that the highly contractile property of C2C12 cells effectively ameliorates the relatively poor HSMM contractile capability and that this is achieved as a consequence of the cells fusing with each other, thereby endowing the hybrid myotubes with EPS-evoked contractile activity.

### Distinguishing between human and mouse myokine expressions by hybrid myotubes

**Figure 3:**
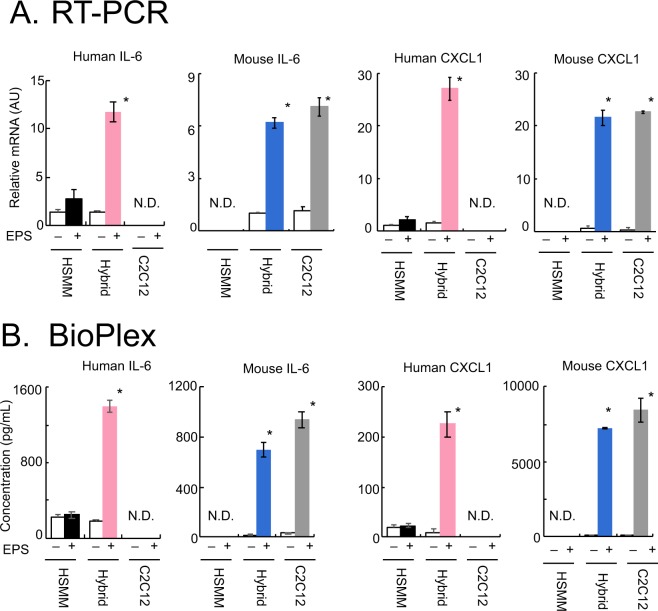
Contraction-inducible upregulation of the expressions and secretions of myokines. After 7–8 days of differentiation, the differentiated myotubes originating from human samples alone (HSMM), human-origin and mouse-origin cells in a mixture (HSMM + C2C12), and murine C2C12 cells alone were treated with or without EPS at 20 V/25 mm, 1 Hz, 4-ms duration for 16 h. (**A**) Total RNA was extracted and the relative abundances of mRNAs for mouse IL-6 and CXCL1 as well as human IL-6 and CXCL1 were evaluated by real-time PCR analysis. Data normalized using mouse GAPDH or human RPLP0 transcripts were averaged over 3 independent experiments (*P < 0.05). (**B**) Conditioned media were collected and concentrations of human IL-6 and CXCL1 as well as mouse IL-6 and CXCL1 were evaluated employing the BioPlex assay (*P < 0.05, n = 3).

We next attempted to evaluate the effects of EPS-evoked contraction on myokine expressions including those of IL-6 and CXCL1 by hybrid myotubes using RT-PCR analysis (Fig. 3A) and the BioPlex assay (Fig. 3B). Given that hybrid myotubes contain both human-derived and mouse-derived nuclei, species-specific PCR primers were designed to separately evaluate the mRNAs of IL-6 and CXCL1 originating from either human or mouse nuclei. As reflected by the poor contractile activity of the myotubes of HSMM alone (Fig. 2), human IL-6 and CXCL1 mRNA expressions were both minimally upregulated by EPS treatment (Fig. 3A, *black bars*). In contrast, hybrid myotubes exhibited significantly elevated expressions of mRNAs for both human IL-6 and CXCL1 in response to EPS-evoked contraction (*pink bars*). As expected, mouse IL-6 and CXCL1 mRNAs were highly upregulated under both hybrid myotube (*blue bars*) and C2C12 alone (*grey bars*) conditions after EPS treatment.

The BioPlex assay further confirmed the RT-PCR results showing human IL-6 and CXCL1 proteins to be highly secreted by contractile hybrid myotubes (Fig. 3B, *pink bars*), but not by myotubes of HSMM alone (*black bars*), after ESP treatment. High levels of mouse IL-6 and CXCL1 were detected in both hybrid myotubes (*blue bars*) and C2C12 myotubes (*grey bars*) after EPS treatment. It should be noted that the BioPlex assay does not show cross-reactions between cells with human versus mouse origins.

Together, these results clearly demonstrated that human and mouse IL-6 and CXCL1 could be individually evaluated by both RT-PCR and the BioPlex assay with species-specific analysis platforms even in the presence of both species in a culture, and thus provide compelling evidence that hybrid myotubes are responsive to EPS-evoked contraction in terms of exercise-induced myokine upregulation of both mRNA and protein secretion. These findings are consistent with those obtained in highly developed contractile myotubes derived from C2C12 alone^1^.

### Hybrid myotubes of primary satellite cells derived from human patient and C2C12 cells

**Figure 4:**
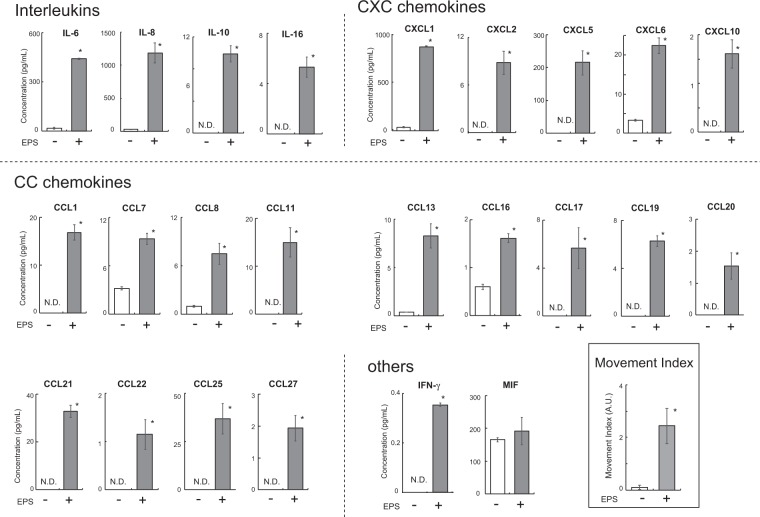
Secretion of contraction-inducible myokines from hybrid myotubes comprised of C2C12 cells and human muscle cells obtained from a biopsy sample. After 7–8 days of differentiation, the differentiated myotubes originating from murine C2C12 cells and human primary myoblasts obtained from a biopsy sample (seeded in the 4 individual wells of 2 distinct culture plates) were treated with (*n* = 4) or without (*n* = 4) EPS at 20 V/25 mm, 1 Hz, 4-ms duration for 16 h. Conditioned media were collected and subjected to the 40-Plex BioPlex assay (*P < 0.05, n = 4). Two independent experiments using the same cell stock sample were performed, and representative results are shown. The Movement Index is also shown in the BOX.

In order to further validate the practicality of our hybrid myotubes, we finally established an “*in vitro* Exercise model” using hybrid myotubes composed of murine C2C12 cells and primary human myoblasts (activated satellite cells) obtained from intact subscapularis muscle of patient who underwent arthroscopic RCT repair surgery. Employing the BioPlex assay (human chemokine 40-plex panel, BioRad Laboratory, Inc.), we measured the amounts of myokines secreted into the conditioned medium after 16 hrs of EPS treatment. The hybrid myotubes exhibited very high levels of myokine (chemokines) secretion including interleukins (IL-6, IL-8, IL-10, and IL16), CXC chemokines (CXCL1, CXCL2, CXCL5, CXCL6, CXCL10), CC chemokines (CCL1, CCL2, CCL7, CCL8, CCL11, CCL13, CCL16, CCL17, CCL19, CCL20, CCL21, CCL22, CCL25, CCL27), and IFN-γ in response to EPS-evoked contractile activity (Fig. 4). MIF (macrophage migration inhibitory factor) was not upregulated by EPS treatment. IL-1β, IL-2, IL4, CXCL9, CXCL11, CXCL13, CCL15, CCL24 CCL16, GM-CSF and TNF-α were not detected, regardless of whether or not EPS treatment was administered (data not shown). It should be noted that in order to assure absolutely no cross-reactivity of murine chemokines in mixed cultures, conditioned media obtained from the C2C12 myotube alone group were separately measured employing the same 40-plex panel, and three chemokines (CX3CL1, CCL3, and CCL12) which emerged as being cross-reactive were excluded (data not shown).

## Discussion

The present studies were conducted with the aim of establishing an “*in vitro* exercise model” using human-mouse hybrid myotubes. Our goal was to maximize the use of valuable human muscle cells obtained from human subjects, such as clinical biopsy specimens, allowing us to acquire important functional properties of muscle cells, particularly contraction-inducible biological responses as well as contractility development. Our system may also shed light on pathological impairments affecting these functions and processes. Although caution is necessary when interpreting data obtained using these hybrid myotubes derived from two different species, our present results provide evidence that inadequacies inherent to primary human muscle cells are effectively overcome by fusing them with murine C2C12 cells which support contractility development (Fig. 2), which may further expand the usefulness of the “*in vitro* exercise model”^1^.

In the present study, we carefully evaluated the status of myogenic fusion between human-origin and mouse-origin muscle cells and found that they had indeed fused with each other to form multi-nucleated hybrid myotubes harboring nuclei derived from the cells of both species (Fig. 1), though the proportion of human nuclei comprised only approximately 15% in the hybrid myotubes. Thus, human-mouse hybrid myotubes, but not human alone or mouse alone myotubes of each individual species under co-cultured conditions, developed contractility together in response to EPS (Fig. 2). Nevertheless, the contractile activity of the hybrid myotubes appears to rely mainly on the properties originating from C2C12. Moreover, the contractile human-mouse hybrid myotubes subsequently exhibited contraction-dependent biological responses including expressions of human myokine mRNAs (Fig. 3A) and actual secretion of myokines (Figs 3B and 4) derived from the human-origin constituents in addition to those of mouse-origin. Notably, despite the smaller populations of human-derived elements within the hybrid myotubes, the human nuclei of these hybrid myotubes were highly responsive to the EPS-evoked contractile activity evoking remarkable upregulations of myokines. This property provides a major research advantage in that it allows us to minimize the number of human muscle cells required from biopsy samples, a very limited resource, for evaluating the contraction-responsive transcriptional ability of human nuclei.

On the other hand, unlike the hybrid myotubes and C2C12 myotubes, human myotubes exhibited very poor contractility accompanied by very little upregulation of myokines in response to EPS, though they showed myogenic differentiation sufficient to form multi-nucleated myotubes expressing caveolin-3, a differentiation marker^23^ (Fig. 1A). One plausible explanation for the limited contractility of human cell-alone myotubes is that these myotubes often display a relatively flattened and spread-out morphology, associated with tight adhesion to the stiff substratum of the culture plate, while C2C12 myotubes formed slender tube-like structures that are apparently ideal for producing contractility even when the same conventional culture apparatus is used. In this regard, like some human myotubes, rat L6 myotubes exhibiting the flattened and spread-out morphology also failed to develop contractile activity under the same EPS culture conditions (data no shown). In addition, the inoculated cell-density and myoblast growth capability might also be important factors since inoculation at a cell density ~5 times higher (6.25 × 10^5^ cells/well of the 8-well plate), which results in 80–90% confluence by the day after seeding, eventually resulted in better contractility of the human myotubes (Supplementary Fig. S1). Thus, we can reasonably speculate that advantageous morphological and adhesive features of C2C12 cells would overcome some of the shortcomings of human cells, yielding vigorous contractility development of human-mouse hybrid myotubes under the culture conditions used in this study. Consistent with this interpretation, hybrid myotubes comprised of human-mouse^24^ and human-cat^25^ cells have also been studied, and malfunctions of human-origin muscle cells such as dystrophin-deficiency could reportedly be partially compensated for by forming hybrid myotubes with the non-human muscle cells.

RT-PCR and BioPlex analyses capable of distinguishing between species clearly demonstrated that EPS-evoked contractile activity remarkably upregulated the mRNAs of myokines from nuclei of both human-origin and mouse-origin constituents in the hybrid myotubes, resulting in significant increases in the secretions of both species’ myokines (IL-6 and CXCL1) into the medium (Fig. 3). Considering the lower proportions of human-derived elements in the hybrid myotubes, human nuclei in the heterokaryon appear to be highly responsive to intracellular signals evoked by the contractile activity in terms of their myokine gene expressions. In contrast, reflecting their poor contractile activity, human-origin alone myotubes showed no contraction-inducible myokine upregulation under the culture conditions used in this study. These results suggest that actual contractile activity, i.e. not just the Ca^2+^ transients evoked by EPS, is required to adequately upregulate these myokines (i.e. IL-6 and CXCL1) and that the actual contractile activity endowed by the fused C2C12 cells may comprehensively evoke contraction-inducible gene expressions from human-origin nuclei of the hybrid myotubes.

Hybrid myotubes arising from primary human myoblasts obtained from a biopsy sample and murine C2C12 cells exhibited marked secretions of numerous human myokines in response to EPS-evoked contractile activity (Fig. 4). Among a total of 40 analytes of human cytokines, an unexpectedly large number of secreted factors, including 4 interleukins, 5 CXC chemokines, 14 CC chemokines and IFN-γ, emerged as being highly accumulated in the conditioned medium only after EPS-evoked contraction. Unlike the *in vivo* state, there are only muscle cells in this *in vitro* exercise model system, such that these soluble factors are undeniably secreted from the hybrid myotubes in response to EPS-evoked contractile activity. While some of these factors have already been well recognized as myokines (and also deemed to be exercise factors)^13,26,27^, our data demonstrate only that the human-derived elements in the hybrid myotubes have the capability of upregulating these human factors in response to EPS-evoked contractile activity. The design of this study did not allow us to directly examine the physiological relevance of our findings and we cannot rule out the possibility that murine-derived C2C12 cells may have influenced the contraction-inducible upregulation of the secreted human factors. Future studies are clearly warranted to determine whether these secreted factors, as identified in the *in vitro* exercise model using hybrid myotubes, are indeed upregulated in the working skeletal muscles of humans and mice *in vivo* in response to exercise.

In summary, we established an “*in vitro* exercise model” using human and murine hybrid myotubes. This model has the potential to be a valuable alternative tool for analyzing contractility and its associated biological responses in human-origin muscle cells, allowing researchers to optimize the use of human muscle cells obtained from biopsy samples.

## Supplementary information

Supplemental Figure S1

Supplementary Movie S1

Supplementary Movie S2

Supplementary Movie S3
