# Supplementary material for: In vitro exercise model using contractile human and mouse hybrid myotubes
Source: Sci Rep. 2019 Aug 15;9:11914. doi: 10.1038/s41598-019-48316-9 (PMC6695424; doi:10.1038/s41598-019-48316-9)
Supplement: Supplementary file 1 — Supplemental Figure S1 [file 41598_2019_48316_MOESM1_ESM.docx]

***In vitro* exercise model using contractile human and mouse hybrid myotubes**

**Weijian Chen, Mazvita R. Nyasha, Masashi Koide,**

**Masahiro Tsuchiya, Naoki Suzuki, Yoshihiro Hagiwara,**

**Masashi Aoki, and Makoto Kanzaki**

**Supplemental Figure S1**

HSMM cells were seeded at a density of 6.25 x 105 cells/well (5-fold higher density) in 3 mL of growth medium in 8-well plates. Two days after plating, differentiation was induced by switching the growth medium to differentiation medium, and the differentiation medium was changed every 24 h during 7 days of differentiation. Differentiated myotubes were placed in a C-Dish and then treated with EPS (1 Hz, 4-ms, 20 V/25 mm) for 16 h as described in the Materials and Methods. Movies of myotubes were taken during EPS at the last 15 min. of the total EPS treatment, and movement index was evaluated as described in the Materials and Methods. Three independent experiments were performed, and the summarized graph is presented (*n*=3; * p<0.05).
